# EEG Neurofeedback Is Under Strong Control of Psychosocial Factors

**DOI:** 10.1007/s10484-018-9407-3

**Published:** 2018-08-04

**Authors:** Guilherme Wood, Silvia Erika Kober

**Affiliations:** 1 Institute of Psychology, Karl-Franzens-University of Graz, Universitaetsplatz 2, 8010 Graz, Austria; 2 BioTechMed, Graz, Austria

**Keywords:** SMR up-regulation, Psychosocial effects, Neurofeedback, Mindfulness, Locus of control in dealing with technology

## Abstract

Recently, a deep impact of psychosocial effects on the outcomes of neurofeedback training was suggested. Previous findings point out an association between locus of control in dealing with technology and the individual ability to up-regulate the sensorimotor rhythm (12–15 Hz) in the EEG. Since the antecedents of locus of control in dealing with technology differ between males and females, we have investigated the effect of sex of participant and experimenter on the outcomes of neurofeedback training. Mindfulness and SMR baseline power also were assessed as possible confounding variables. Undergraduate psychology students (n = 142) took part in a single session of neurofeedback training conducted by either male or female experimenters. Male participants as well as those female participants instructed by male experimenters were able to upregulate SMR, while female participants trained by female experimenters were not. A strong positive correlation between training outcomes and locus of control in dealing with technology was observed only in the female participants trained by female experimenters. These results are suggestive about the impact of psychosocial factors—particularly gender-related effects—on neurofeedback training outcomes and the urgent need to document it in neurofeedback studies.

## Introduction

Neurofeedback is a technique to achieve control of specific brain signals by means of feedback training. Neurofeedback learning outcomes are usually believed to recruit basic mechanisms of skill learning (Sitaram et al. 2017) and be largely independent of consciousness and verbal instructions (Birbaumer et al. 2013). Over the years, some authors caught attention to the role of higher cognitive processes (Strehl 2014; Wood et al. 2014) in neurofeedback learning, but literature review reveals only a few studies directly investigating how these processes determine neurofeedback training outcomes (Alkoby et al. 2017). Other authors argued that outcomes of neurofeedback training are probably not as much driven by basic mechanisms of skill learning (Thibault et al. 2017), as by placebo or other expectancy and psychosocial effects (Thibault et al. 2016; Thibault and Raz 2017).

Some studies report an effect of psychosocial factors on the ability to self-regulate brain activity. Burde and Blankertz (2006) assessed locus of control in dealing with technology as indexed by the KUT (control beliefs in dealing with technology; Beier 2004) influences the ability to down-regulate the sensorimotor rhythm of EEG (12–15 Hz, henceforth SMR) in healthy participants during a motor imagery task. These authors reported a positive correlation between locus of control in dealing with technology and the down-regulation of the SMR rhythm. Moreover, Witte et al. (2013) investigated the association between locus of control in dealing with technology and the ability to upregulate SMR power through neurofeedback training. A strong negative correlation between locus of control in dealing with technology and the ability to upregulate SMR was observed after ten training sessions. Together, these results are suggestive about the existence of an association between locus of control in dealing with technology and the ability to both up- and down-regulate the SMR. The mechanisms responsible for this association remain nevertheless elusive. Witte et al. (2013) argued that stronger locus of control in dealing with technology may lead to an increase of conscious effort to regulate brain signals during training. Since the regulation of brain signals depends mainly on non-conscious associative learning mechanisms (Birbaumer et al. 2013; Gruzelier 2014; Sitaram et al. 2017), such increase of conscious effort should be counterproductive and might reduce the responsivity to neurofeedback training programs.

Interestingly, locus of control in dealing with technology is also associated with gender roles. In a study examining the effect of gender roles on technology self-efficacy in undergraduate psychology students, a positive correlation between masculinity and technology self-efficacy was observed (Huffman et al. 2013). In another study, Saleem et al. (2011) found out that the antecedents of computer self-efficacy are different in male and female students. Accordingly, the correlations between locus of control in dealing with technology and the ability to up-regulate the SMR may be gender specific. Unfortunately, earlier neurofeedback studies on the topic examined too few participants to be informative on this respect (Burde and Blankertz 2006; Witte et al. 2013).

Interactions between the gender of participant and experimenter are well known and can determine the intensity and direction of gender effects. For instance, Kállai et al. (2004) reported a significant interaction of experimenter gender and participant gender on pain tolerance. These authors observed longer tolerance for pain when they were tested by an experimenter of the opposite sex as well as a significant main effect for experimenter gender, which indicates higher pain intensities for participants tested by female experimenters. These findings have been confirmed by meta-analytical approaches (Alabas et al. 2012). Moreover, Aslaksen et al. (2007) report significant interactions between experimenter gender and participant gender on pain intensity and arousal, but no interactions in the physiological data. This interesting finding indicates that the lower pain report in male subjects to female experimenters was not mediated by changes in autonomic parameters but rather by psychosocial factors. Therefore, it is not clear whether gender of participant and experimenter have an impact on learning observed during neurofeedback training. On the one side, gender has a selective impact on locus of control in dealing with technology, which themselves have an impact on SMR neurofeedback. Accordingly, at least an indirect effect of gender on neurofeedback outcomes may exist. As indicated by studies on tolerance for pain, physiological responses may not necessarily be affected by main- and interaction effects involving gender (Aslaksen et al. 2007). Therefore, gender effects on neurofeedback may also be psychosocial and not physiological. On the other side, neuroscientific techniques are able to elicit strong expectation effects even in well-educated undergraduate students (Ali et al. 2014), and these expectations may trigger specific vigilance and arousal reactions (Schwarz et al. 2016), which may indeed interfere with the (electro)physiological basis of SMR up-regulation training. Accordingly, gender may lead to specific responses to SMR neurofeedback training.

In the present study, the effects of sex of participant, sex of experimenter, as well as the role of locus of control in dealing with technology will be investigated. In previous studies, mindfulness (Kikkert 2015; Kober et al. 2017b) and SMR baseline power (Reichert et al. 2015) have been related to SMR up-regulation learning. Higher mindfulness scores predict a higher ability not only to up-regulate the SMR rhythm (Kober et al. 2017b), but also other EEG rhythms (Kikkert 2015). Moreover, different studies reported that SMR baseline power is associated with the ability to regulate the SMR both up (Reichert et al. 2015) and down (Blankertz et al. 2010). Although the purpose of the present study is not to investigate further the effects of mindfulness and SMR baseline power on neurofeedback training outcomes, their impact will be measured and controlled statistically in the experimental design.

## Methods

### Participants

Undergraduate students took part in this study (*n* = 142, 48 females, mean age = 23 years, *SD* = 3.2 years, see Table 1 for more details). Participants received time credits for their participation in the study. They were informed about their right to interrupt participation in the study at any time without the specification of particular reasons. Data were collected by 13 undergraduate students (6 female, mean age = 23 years, *SD* = 2 years) acting as experimenters, who were not informed about the investigation of gender effects. Experimenters trained between 8 and 14 participants of both sexes (average = 11, *SD* = 2). Data of further 14 participants were deleted from the data basis because of faulty EEG recordings or failure to fill one or more questionnaires. The study was approved by the local ethics committee and conducted in conformity with the conference of Helsinki.

**Table 1:** Distribution of age, baseline SMR, locus of control in dealing with technology (KUT), mindfulness, and number of responders per group

Group	Age (years)	Baseline SMR (µV^2^)	Control beliefs	Mindfulness	No. responders
Female_exp_–female_partic_ n = 19	23 (0.72)	2.49 (0.41)	30 (1.52)	39 (1.29)	8/19
Female_exp_–male_partic_ n = 57	23 (0.42)	2.84 (0.24)	30 (0.88)	38 (0.75)	36/57
Male_exp_–female_partic_ n = 29	25 (0.58)	2.75 (0.33)	31 (1.23)	39 (1.04)	21/29
Male_exp_–male_partic_ n = 37	23 (0.52)	2.46 (0.29)	28 (1.09)	39 (0.92)	26/37

### Freiburg Mindfulness Inventory (FMI, Short Version, Walach et al. 2006)

This 14 items scale is a one-dimensional, semantically robust, and psychometrically stable (Cronbach’s alpha = 0.86) measurement of mindfulness. Items are constructed in a 4-point Likert scale rating the frequency of subjective experiences as “rarely”, “occasionally”, “fairly often”, or “almost always”. Typical items are “I see my mistakes and difficulties without judging them” and “I watch my feelings without getting lost in them”. Correlation with other relevant constructs (self-awareness, dissociation, global severity index, meditation experience in years) have been observed, which lends construct validity to the scale (Walach et al. 2006). Responses to single items are coded as numbers between 1 and 4 and added to form the total score. Higher scores in the FMI indicate higher levels of mindfulness.

### Perceived Locus of Control in Dealing with Technology Questionnaire (KUT)

The locus of control in dealing with technology was assessed in the context of dealing with technology by the KUT questionnaire (Beier 1999, 2004). Typical items are “I feel helpless when dealing with technical devices and prefer to keep away from them” and “I enjoy very much solving technical problems”. This one dimensional construct of locus of control in dealing with technology is a subjective 5-point Likert scale rating that considers actual technologic biography in eight items (range of total score 8–40). The questionnaire is available in German, and has a high reliability and has been used in other brain-computer interface studies (Burde and Blankertz 2006; Witte et al. 2013). Items are numerically coded to a value between 1 and 5. Half the items have to be recoded before adding the individual items to obtain the total score. Higher scores in the KUT indicate stronger beliefs of control in dealing with technology.

### EEG Recording/Neurofeedback Training

**Fig. 1:**
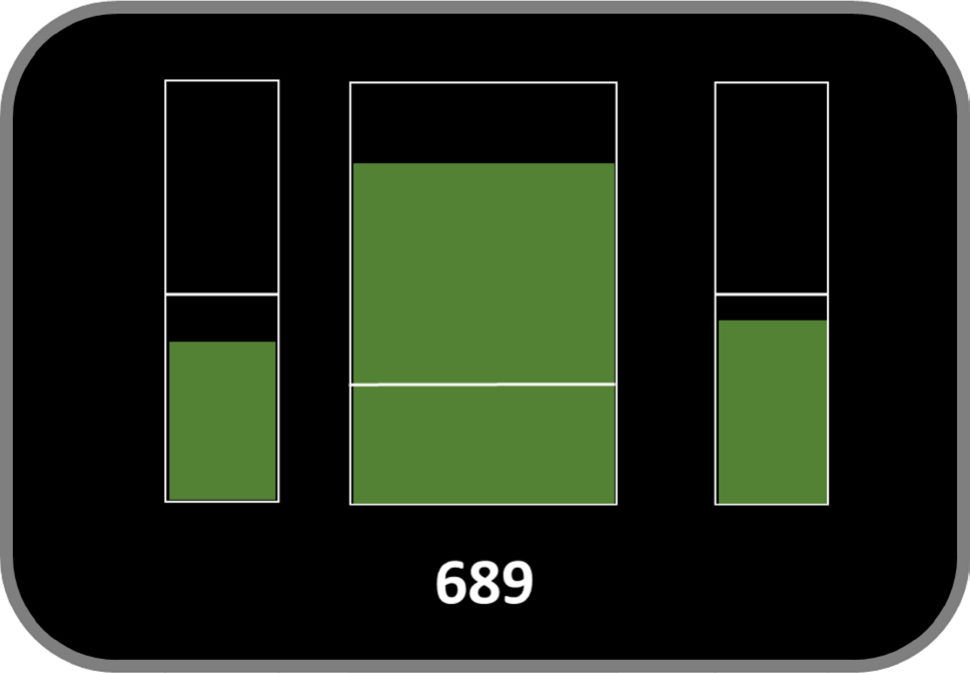
Example of the neurofeedback display employed in the present study. The middle bar depicts the SMR power, the left one the oscillations in theta frequency (4–8 Hz) and the right one oscillations in beta frequency (21–35 Hz). The white horizontal lines represent the thresholds. Only when power in theta and beta frequency bands were below and the power in SMR was above their respective thresholds, positive feedback was presented (bars turned green and points were added to the total score). Otherwise, bars indicating undesired power levels turned red and no point was added to the total score

All participants performed one single NF training session of about 45 min. For neurofeedback generation, signals obtained at Cz were used. EEG was recorded with a sampling frequency of 256 Hz, the ground was located at the right mastoid; the reference electrode was placed at the left mastoid. One EOG channel was placed at the left eye. The signals were amplified by a 10-channel system (NeXus-10 MKII, Mind Media BV). The NF training paradigms were generated by using the software BioTrace+ (Mind Media BV). Audio-visual feedback was provided by a moving bar in the center of the feedback screen depicting the amplitude of SMR (12–15 Hz) activity and a midi-tone feedback, whenever the moving bar preceded an individually defined threshold value. Two additional smaller bars on the left and on the right side of the screen depicted EOG artefacts (4–8 Hz, theta) and muscle artefacts (21–35 Hz, beta), respectively. This procedure controls for the artificial increase in SMR power by mean of artifacts (Kober et al. 2015, 2017a). The three EEG frequency bands (SMR, theta, and beta) were filtered online in the respective frequency ranges using IIR butterworth (3rd order) filters. A three minutes baseline run at the beginning of each training session, in which participants saw the moving bars but were instructed to relax themselves while receiving no reward, was used to calculate the individually defined thresholds for the subsequent feedback runs (Fig. 1).

For the SMR bar, the mean amplitude of SMR activity assessed during the baseline run was used as threshold. This threshold was adapted after each run based on the mean SMR activity of previous runs. For the two control bars, the mean activity during the baseline run + 1 SD was used as threshold value. After the baseline run, six 3 min long feedback runs were performed, in which participants were instructed to increase the bar in the middle of the screen, while keeping the artefact bars on the left and right side of the screen below their thresholds.

Participants’ task was to increase the height of the central bar and at the same time keep the two smaller bars as low as possible. To facilitate the recognition of current performance, the central bar changed color from red to green whenever a pre-defined threshold was reached and participants received an additional rewarding feedback of one earned point per 250 ms above threshold.

### Statistical Analysis

EEG data were inspected by SEK. Ocular artifacts such as eye blinks were manually rejected by visual inspection based on activity of the EOG channel. After ocular artifact correction, automated rejection of other EEG artifacts (e.g. muscles) was performed (criteria for rejection: > 50.00 µV voltage step per sampling point, absolute voltage value > ± 120.00 µV). Power in different frequency bandy (SMR 12–15 Hz, theta 4–8 Hz, beta 21–35 Hz) were extracted by means of complex demodulation (Brain Products GmbH 2009). Absolute power values in the three different frequency bands were z-transformed for each individual to be able to represent the neurofeedback learning effect in a scale common to all participants.

Training effects were evaluated using a linear mixed-effects model in which training runs (1–7 for baseline + 6 training runs) was modelled as a linear contrast (see Pinhas et al. 2012 for the rationale), sex of experimenter and sex of participant as fixed effects, individual subjects and individual experimenter as two random-effects. Importantly, individual subjects (142 levels) were nested in the individual experimenters (13 levels) in the model. Higher SMR power levels at the baseline (Reichert et al. 2015), higher mindfulness scores (Kikkert 2015; Kober et al. 2017b), as well as lower scores in locus of control in dealing with technology (Witte et al. 2013) have been related to higher ability to up-regulate the SMR. Therefore, these values were entered as covariates in the mixed-effects model designed to evaluate the effects of gender on SMR NF training. Calculations were performed in R using the libraries lme4, lmerTest, and phia to calculate, respectively, model parameters, statistics with the appropriate number of degrees of freedom and post-hoc contrasts. F-values, degrees of freedom and MSE were calculated using the Satterthwaite method. p-values were adjusted for multiple comparisons using the Holm method.

## Results

Baseline SMR power, locus of control in dealing with technology (KUT), and mindfulness were compared in separated 2 × 2 ANOVA models having sex of experimenter and sex of participant as fixed-effects factors. Analyzes revealed no significant main- or interaction effects (all p > 0.05). Group-specific average and standard deviation values of these variables are depicted in Table 1. Because of these results, in all further analyses we used the individual z-transform SMR power values as de-pendent variable.

### NF Outcomes and Their Determinants

**Fig. 2:**
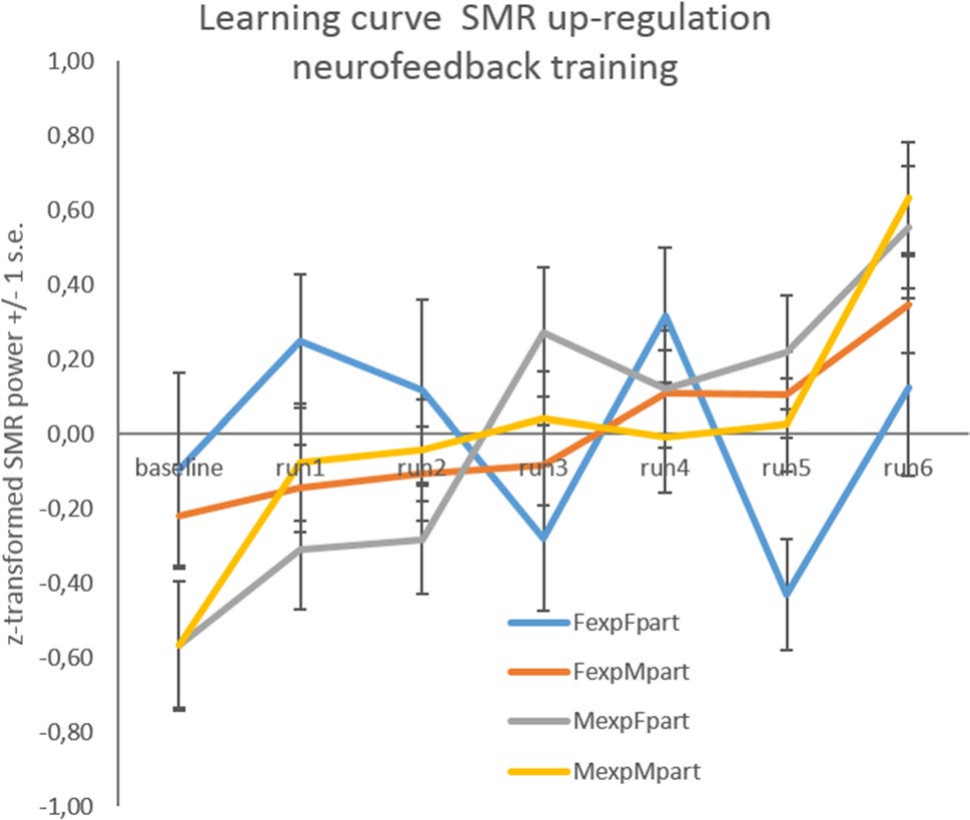
Learning curves (means and se of z-transformed SMR power) observed during one session of SMR up-regulation neurofeedback training. Female_exp_Female_part_, Female experimenter–female participant, Female_exp_Male_part_: Female experimenter–male participant, Male_exp_Female_part_: Male experimenter–female participant, Female_exp_Male_part_: Male experimenter–Male participant

Learning effects are depicted per group in Fig. 2. The SMR power increased linearly in function of training runs (*F*(1, 132) = 27.6; *MSE* = 18.5, *p* = 5.76 × 10^− 7^; *η*^*2*^ = 0.18). This is indicative of a strong learning effect during neurofeedback. The learning effect was under influence of the covariates mindfulness (*F*(1, 132) = 4.3; *MSE* = 2.8; *p* = 0.04; *η*^*2*^ = 0.03) and SMR baseline power (*F*(1, 132) = 4.0; *MSE* = 2.7; *p* = 0.05; *η*^*2*^ = 0.03). The weak correlations individual learning effect vs. mindfulness (*r*(142) = 0.17; *p* = 0.04) and individual learning effect vs. SMR baseline power (r(142) = − 0.18; *p* = 0.04) were observed.

**Fig. 3:**
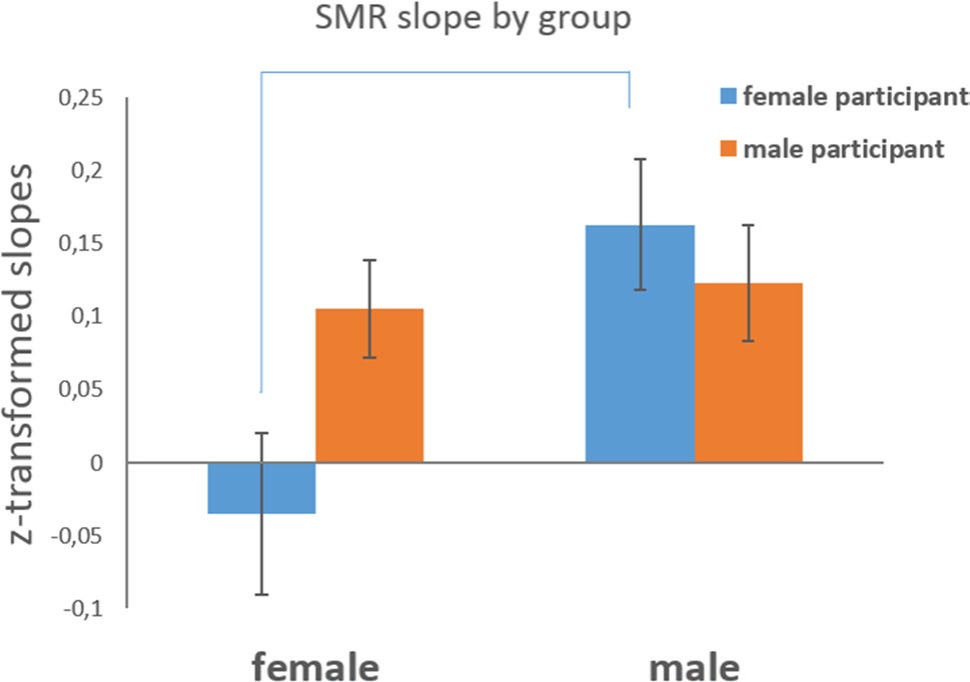
Z-transformed SMR slopes averaged per group. The connecting line represents a significant difference between groups (p < 0.05, corrected for multiple comparisons)

The learning effect interacted with the sex of the experimenter (*F*(1, 132) = 6.0; *MSE* = 4.0; *p* = 0.02; *η*^*2*^ = 0.04). The triple interaction sex of experimenter vs. sex of participant vs. learning effect just failed to reach significance (*F*(1, 132) = 3.6; *MSE* = 2.4; *p* = 0.06; *η*^*2*^ = 0.03), but the quadruple interaction sex of experimenter versus sex of participant versus learning effect versus locus of control in dealing with technology was highly significant (*F*(1, 132) = 9.3; *MSE* = 6.2; *p* = 0.003; *η*^*2*^ = 0.07). To understand this interaction better, we examined the correlation between locus of control in dealing with technology and the learning effect in the four groups of participants. A strong positive correlation was obtained in the Female_exp_–Female_partic_ group (*r*(19) = 0.79), while no correlation was obtained in the other three groups (all *r*’s < ± 0.1). Moreover, we compared pairwise the learning effect across groups using Holm adjusted post-hoc contrasts. Only the comparison between Female_exp_–Female_partic_ and Male_exp_–Female_partic_ reached significance (*F*(1, 110) = 8.0; *sum of squares* = 0.45; *p* = 0.01; *η*^*2*^ = 0.07). As depicted in Fig. 3, female participants trained by female experimenters learned significantly less than their counterparts trained by male experimenters. The comparison of individual learning slopes with 0 revealed no evidence of learning in the group Female_exp_-Female_partic_, (*b* = − 0.018, 95% CI (− 0.13, 0.09); *t*(18) = − 0.34, *p* = 0.74). In all other groups, the learning effect was consistently larger than 0 [Female_exp_–Male_partic_: *b* = 0.086, 95% CI (0.018, 0.15); Male_exp_–Female_partic_: *b* = 0.172, 95% CI (0.08, 0.26); Male_exp_–Male_partic_: *b* = 0.14, 95% CI (0.056, 0.22)], and statistically significant (Female_exp_–Male_partic_: *t*(56) = 2.54, *p* = 0.014; Male_exp_–Female_partic_: *t*(28) = 3.89, *p* = 0.001; Male_exp_–Male_partic_: *t*(37) = 3.44, *p* = 0.001). In summary, the quadruple interaction is characterized by (i) absence of learning and (ii) presence of a strong positive correlation between locus of control in dealing with technology and the learning effect in the Female_exp_–Female_partic_only, but not the other groups.

## Discussion

In the present study, we disclosed effects of gender on the learning outcomes observed during a session of SMR neurofeedback training. The combination female experimenter–female participant hampered the training outcomes of the last, so that no learning effect was observed in this group. Sex-related effects remained significant after removal of the effects of experimenter, participant’s mindfulness, and participant’s baseline SMR power. Moreover, an association between locus of control in dealing with technology and individual learning outcomes was observed only when female participants were trained by female experimenters. In the following, these results will be discussed in more detail.

Higher levels of mindfulness were associated with higher ability to up-regulate the SMR. These results corroborate previous studies showing a positive effect of mindfulness on the ability to modulate brain signals in NF training (Kikkert 2015; Kober et al. 2017b). Interestingly, mindfulness did not differ between female and male participants in the present study. Moreover, analyses revealed that the effect of mindfulness on the ability to up-regulate SMR remains significant after removing the effects of locus of control in dealing with technology, experimenter, and SMR baseline power. Therefore, a specific albeit weak positive impact of mindfulness on the ability to up-regulate SMR seems to be detectable even when neurofeedback training is limited to a single session of about half an hour. The reasons why the estimate of the impact of mindfulness on neurofeedback training seems to be stronger in previous studies seem to be twofold. The strong positive correlation observed by Kikkert (2015) was calculated after participants trained for several sessions, what reveals more clearly individual differences between responders and non-responders. In that study, participants trained theta inhibition and beta enhancement and not SMR up-regulation, so that the correlation with mindfulness reported by Kikkert (2015) cannot be directly compared with our results. Moreover, the statistical size of the positive effect of mindfulness as observed by Kober et al. (2017b) was obtained in a comparison of a group of highly spiritual- and strongly mindful-participants with average controls. For these reasons, one may argue that the positive association between mindfulness and the ability to up-regulate SMR observed in the present study is in line with the literature (Kikkert 2015; Kober et al. 2017b). The ability to focus in the present moment and empty the mind from intrusive thoughts seems to be advantageous for SMR up-regulation training even when the amount of training is very limited (Kober et al. 2013, 2017b).

A weak negative correlation between SMR baseline power and SMR slopes was observed. These results seem to contradict previous studies indicating a positive association between SMR regulation and SMR power at resting-states (Blankertz et al. 2010; Reichert et al. 2015). In contrast to the present study, evidence that higher SMR resting-states power are associated with better up-regulation of SMR was obtained in training programs involving several training sessions (Reichert et al. 2015). Moreover, the disparity between the present results and those reported by Reichert et al. (2015) may be due to differences in how SMR baseline power estimates were obtained in both studies. Reichert et al. (2015) estimated the SMR baseline power based on a 2 min resting-states measurement with a blank computer screen. In the present study, SMR baseline power was obtained during a 2 min measurement in which a moving bar was visible. Given that the measurements of SMR baseline power are not directly comparable with those employed by Reichert et al. (2015; see also Blankertz et al. 2010), the present results are not conclusive regarding possible advantages or disadvantages of having higher SMR baseline power for SMR up-regulation. Despite these limitations, the present results do not jeopardize the interpretation of the role of sex and locus of control in dealing with technology in the up-regulation of SMR. Since SMR baseline power was comparable across all four groups, gender effects on the outcomes of neurofeedback training cannot be attributed to a general inflation or suppression of SMR power in some of the groups.

The most important results of the present study are the absence of SMR upregulation observed when female participants were trained by female experimenters. Since we controlled for the effect of individual experimenters in the statistical analyses by means of a random-effect with 13 levels, the sex differences observed in the present study are not attributable to the personal influence of single individuals, but seem to have a broader scope. Moreover, not only the regression slope representing the learning effect was not significant, but also the number of responders in this group was lower than expected by chance when considering the rest of participants of the present study as a reference. Also when taking other studies as a reference to estimate the number of responders (i.e. 75% in Kober et al. 2015), the number of responders in the group of female participants trained by female experimenters is also low. In contrast, the three other groups showed learning outcomes largely comparable among themselves as well as with previous studies (Kober et al. 2015). Since the effect of individual experimenter was also considered in the statistical models, these results indicate that the combination of female experimenter and female participant is unfavorable for the up-regulation of the SMR. Importantly, a strong positive correlation with the KUT was observed only in the group of female participants trained by female experimenters. This contradicts previous findings in two different ways. First, Witte et al. (2013) only observed correlations after 10 sessions training, not after the very first one. Second, the correlations observed by Witte et al. (2013) were negative. Witte et al. (2013) argued that the negative correlation between locus of control in dealing with technology and SMR upregulation is due to the negative effect of control expectations on neurofeedback learning, for it is based mainly on implicit processes beyond explicit control beliefs and expectations. Since the opposite effect was observed in the present study after a much shorter training, the determinants of a correlation in the two studies are probably not the same. The sex differences observed during neurofeedback learning may have a psychosocial origin and be unrelated to basic mechanisms of implicit learning. One possibility is that asking participants about locus of control in dealing with technology framed the training situation inadvertently by using the specific scale (Schwarz et al. 2016). Considering that locus of control in dealing with technology are associated with personality traits (Saleem et al. 2011) and the subjective norm (Venkatesh and Morris 2000) in female participants only, the interaction between female participant and female experimenter may involve an extra load of expectancy when compared to the interactions involving at least one male individual. This extra load of expectations may consume cognitive resources necessary for neurofeedback training (Schwarz et al. 2016; see also Kober et al. 2013). Considering that even undergraduate students trained in neurosciences may show an implausibly high degree of confidence in the effectiveness of imaging technology (Ali et al. 2014) such as neurofeedback, the emergency of expectancy effects merely by the use of a scale to measure locus of control in dealing with technology is by no means surprising or trivial. To the contrary, they provide evidence that psychosocial factors interfere with neurofeedback training outcomes (Thibault and Raz 2017).

Even if an undesired framing effect was uniquely responsible for the present results, the influence of psychosocial effects on neurofeedback training outcomes will probably not be suppressed by the removal of this control beliefs scale from the test protocol, but may take many other forms, which hitherto have not be investigated empirically (Thibault and Raz 2017). A recent review points out that sex of experimenter as well as sex of participant may be responsible for the lack of replicability of the outcomes of clinical interventions (Chapman et al. 2018). Accordingly, the relevance of our results is diminished, since they indicate that even subtle aspects of the context in which neurofeedback training is performed may elicit specific psychophysiological responses (Schwarz et al. 2016) and change outcomes substantially. Although the effects of sex of participant and experimenter on the individual ability to up-regulate SMR as reported in the present study have only small or moderate statistical size and may be attributable to the framing caused by the employment of a questionnaire of locus of control in dealing with technology, they should not be neglected. First, gender of participants was not assessed directly in the present study, only their biological sex. A more detailed characterization of participants and experimenters regarding the trait masculinity/femininity may reveal more pronounced effects (Bussey and Bandura 1999). Second, the typical design of neurofeedback studies includes only a modest number of participants, which is insufficient to detect the presence of more subtle (but existent) statistical effects. Consequently, the estimates of SMR up-regulation observed in the literature may be biased by systematic sources of heterogeneity that reduce the estimated treatment effect. Third, gender of experimenter and gender of participants should be considered more earnestly in the design of neurofeedback interventions (Chapman et al. 2018). Finally, the existence of a substantial number of non-responders among participants trying to self-regulate their brain activity calls for the development of a model of the determinants of neurofeedback outcomes (Enriquez-Geppert et al. 2017), in which psychosocial effects should be considered as well (Thibault and Raz 2017).
